# PTMs_Closed_Search: Multiple Post-Translational Modification Closed Search Using Reduced Search Space and Transferred FDR

**DOI:** 10.3390/proteomes14010007

**Published:** 2026-02-02

**Authors:** Yury Yu. Strogov, Sergey A. Spirin, Mark V. Ivanov, Maria A. Kulebyakina, Anastasia Yu. Efimenko, Oleg I. Klychnikov

**Affiliations:** 1 Faculty of Bioengineering and Bioinformatics, Lomonosov Moscow State University, Moscow 119991, Russia; yurystrogov@gmail.com; 2 Belozersky Institute of Physico-Chemical Biology, Lomonosov Moscow State University, Moscow 119991, Russia; sas@belozersky.msu.ru; 3 Faculty of Computer Science, Higher School of Economics, 20 Myasnitskaya St., Moscow 101000, Russia; 4 Scientific Research Institute for System Analysis of the National Research Centre Kurchatov Institute, 36 Nakhimovsky pr., bld. 1, Moscow 117218, Russia; 5 V. L. Talrose Institute for Energy Problems of Chemical Physics, N. N. Semenov Federal Research Center for Chemical Physics, Russian Academy of Sciences, 38 Leninsky pr., Bld. 2, Moscow 119334, Russia; markmipt@gmail.com; 6 Medical Research and Educational Institute, Lomonosov Moscow State University, 27/10, Lomonosovskiy Av., Moscow 119234, Russia; m.a.kulebyakina@gmail.com (M.A.K.); efimenkoay@my.msu.ru (A.Y.E.); 7 Faculty of Biology, Lomonosov Moscow State University, Moscow 119991, Russia

**Keywords:** algorithms, closed search, group-specific FDR, post-translational modification

## Abstract

**Background**: Currently, post-translational modification (PTM) search in MS/MS data is performed using either open modification search (OMS) or closed search (CS) algorithms. The OMS method allows for the determination of many PTMs and unknown mass-shifts in one run. In contrast, closed search algorithms are more sensitive but limited in the number of PTMs that can be specified in one search. **Methods**: In this manuscript, we propose an optimized Python algorithm based on the IdentiPy search engine that performs an automated sequential search for each PTM based on previous annotations from public databases and customized protein lists. We also determined the sufficient size of the search space to increase the significance of false discovery rate (FDR) estimation. We modified the FDR calculation algorithm by implementing a spline approximation of the ratio of the modified decoys, and by calculating error propagation to filter out unstable data and determine the cutoff value. **Results**: The results of this pipeline for a test dataset were comparable to previously published data in terms of the number of unmodified peptides and proteins. Additionally, we identified 13 different types of peptide PTMs and achieved an increase in relative protein coverage. Our filtration method based on spline transferred FDR showed a superior number of identified peptides compared to separate FDR. **Conclusions**: Our developed pipeline can be used as a standalone application or as a module of multiple PTM search in data analysis platforms.

## 1. Introduction

Post-translational modifications (PTMs) are key mechanisms for the formation of proteoforms that regulate protein activity, stability, lifetime, subcellular localization, protein–protein interactions, and cell signaling. With over 400 types of PTMs described [1], they impact a wide range of processes in both pro- and eukaryotic cells [2]. Therefore, a broad range of biochemical tools have been developed to study PTMs. These tools include in vivo and in vitro isotopic labeling (e.g., introduction of ^32^P to detect phosphorylation), a wide range of immunochemical methods (which allow the detection of PTMs such as glycosylation, ubiquitination, SUMOylation, nitrosylation, etc.), and mass-spectrometry analysis of entire proteomes to reveal the complete PTM landscape of a cell in vivo [2,3].

Currently, various search engines allow performing either a closed search (CS) or an open modification search (OMS) to identify PTMs. In classical CS algorithms, the key parameter for peptide identification is a narrow precursor mass window and a specified number of variable modifications included in one search [4]. However, CS algorithms have some limitations in PTM searches. In practice, there is only a limited number of different variable modifications that can be set during a single search run. Including a larger set of variable modifications in a search results in a combinatorial explosion of peptide candidates in the search space. This increases search time and leads to a higher number of false-positive identifications, making FDR estimation challenging [5,6,7]. To address the challenges of large-scale searches, a one-by-one algorithm was introduced to identify single nucleotide polymorphisms (SNPs) [8,9,10]. For FDR estimation for PTM, transferred FDR was introduced for PTM-specific FDR estimation [11]. Although this sequential analysis overcomes the combinatorial explosion problem while maintaining the high sensitivity that is an advantage of CS algorithms, the overall execution time increases linearly with the number of PTMs tested against the entire database.

In contrast, OMS facilitates identifying all possible modifications within a wide mass range (e.g., ±500 Da). In its simplest form, the precursor mass tolerance in a protein database search extends to the selected mass range. The peptide modification is then determined by the delta mass of the precursor and the peptide candidate [4,12]. This strategy was implemented in search engines such as MODa [13], PIPI [14], MSFragger [15], and TagGraph [16].

Previous studies have shown that OMS results in a greater number of identifications, while the CS demonstrates higher sensitivity to individual modifications [4,12]. As a result, there is less than 30% overlap between the sets of peptides identified with these two approaches, indicating that they are complementary [12].

In this study, we present pipeline implementation of a sequential closed search for modifications with a PTM-specific FDR implementation. To shorten the execution time of the complete analysis, the “looking under a spotlight” strategy (a fast search against only a list of proteins with annotated PTMs) was compared with a full search (against the full proteome database). We also tested these two strategies in order to check if information was lost when the size of the search database was reduced. The variety of PTMs and the list of proteins to be tested are automatically generated based on the initial standard CS results combined with information from public databases (Uniprot and dbPTM) and custom-made lists. Separate PTM-specific searches are then conducted according to this information. This method of multiple searches and localizations of PTMs helps determine the spectrum of protein proteoforms in a sample with high confidence.

## 2. Materials and Methods

### 2.1. Datasets

The reference datasets used to test the software were obtained from previously published manuscripts. We compared three sets of data [12,17,18]. Two of them came from shotgun screening experiments, and the third came from a spike experiment with known amounts of ubiquitinated proteins.

The first dataset of HEK293 was created by Chick et al. [12]. The 24 raw files were downloaded from ProteomeXchange (PRIDE number PXD001468). This proteome was prepared as follows: HEK293 human cell lysates were denatured with urea, reduced with DTT, and cysteines were alkylated with iodoacetamide; digestion was performed with Lys-C and with trypsin. The resulting peptide samples were fractionated by means of basic pH reversed-phase liquid chromatography into 24 fractions and analyzed by Q-Exactive Orbitrap (Thermo Scientific, San Jose, CA, USA) using 3 h gradients. Peptides were separated using a 3 h gradient of 6 to 30% acetonitrile gradient in 0.125% formic acid with a flow rate of ∼300 nL/min. In each data collection cycle, one full MS scan (300–1500 *m*/*z*) was acquired in the Orbitrap (7 × 10^4^ resolution setting, automatic gain control (AGC) target of 3 × 10^6^), and the top 20 most abundant ions were selected for isolation and fragmentation by HCD. Ions were selected for isolation when their intensity reached a threshold of 500 counts. HCD was performed using a 2 *m*/*z* isolation window, a resolution of 1.75 × 10^4^, an AGC setting of 5 × 10^5^, and a maximum ion accumulation time of 60 ms. Previously selected ions were dynamically excluded for 60 s. More details on sample preparation and LC-MS/MS protocols can be found in the original manuscript [12].

The second validation set consisted of six files from HeLa cell lysates (PRIDE number PXD051723) [17]. HeLa cells were flash-frozen in liquid nitrogen and stored at −80 °C. The cells were lysed in 0.1 M Tris-HCl, pH 7.5, containing 0.1 M dithiothreitol and 4% SDS, incubated at 95 °C for 5 min, sonicated, and clarified by centrifugation. Peptides were separated by reversed-phase liquid chromatography using a multi-hour acetonitrile gradient and analyzed on an LTQ Orbitrap Velos mass spectrometer (Thermo Scientific, San Jose, CA, USA). Mass spectra were acquired in a data-dependent manner, with an automatic switch between MS and MS/MS scans using a top 10 method. MS spectra were acquired in the Orbitrap analyzer, with a mass range of 300–1650 Th and a target value of 10^6^ ions. Peptide fragmentation was performed with the HCD method, and MS/MS spectra were acquired at the target value of 40,000 ions. The ion selection threshold was set to 5000 counts [17].

The third dataset used in this study corresponds to the spike-in benchmark samples (MassIVE MSV000088971) [18]. These samples were used to evaluate the statistical protocol for ubiquitination in a label-free experiment. HEK293 cells were lysed in denaturing buffer (8 M urea, 20 mM HEPES pH 8.0, 1 mM sodium orthovanadate, 2.5 mM sodium pyrophosphate, and 1 mM β-glycerophosphate) and subjected to two rounds of microtip sonication (9 W for 30 s) at a low temperature. Lysates were clarified via high-speed centrifugation (18,000× *g*, 15 min) to remove insoluble material, reduced (4.1 mM dithiothreitol, 60 min at 37 °C), and alkylated (9.1 mM iodoacetamide, 15 min at room temperature). Protein lysate (120 mg) was diluted 4-fold and subjected to overnight enzymatic digestion at 37 °C with a combination of lysyl-endopeptidase (Wako, Osaka, Japan) and sequencing-grade trypsin (Promega, Madison, Wisconsin, USA), both at an enzyme-to-protein ratio of 1:100, the latter of which was added to the sample 3 h post-incubation with the former. Resultant peptides were acidified with 20% TFA to a final concentration of 1%, clarified via high-speed centrifugation (18,000× *g*, 10 min) prior to desalting via Sep-pak C18 solid-phase extraction (Waters, Milford, MA, USA). Spike-in peptides were added prior to proceeding with immunoaffinity enrichment. Samples were analyzed by liquid chromatography tandem mass spectrometry (LC-MS/MS) on an Orbitrap Lumos mass spectrometer (ThermoFisher, San Jose, CA, USA) coupled to a Dionex Ultimate 3000 RSLC (ThermoFisher, San Jose, CA, USA) employing an Aurora Series 25 cm × 75 um I.D. column (IonOpticks, Victoria, Australia). Peptides were separated at a flowrate of 450 nL/minute over a 95 min dual-stage linear gradient whereby solvent B (0.1% FA/98%ACN/2% water) was ramped from 2% to 35% over 85 min and then from 35% to 50% over 10 min with a total run time of 120 min. The mass spectrometer was operated in data-dependent acquisition mode with MS1 precursor ions analyzed in the FT at 240,000 resolution with an AGC target of 10^6^. MS2 was performed in the linear ion trap with a high-energy collision dissociation (HCD) normalized collision energy of 30%, an AGC target of 40,000, an isolation width of 0.7 *m*/*z*, and a 10 s dynamic exclusion window [18].

### 2.2. Access and Implementation

The “ptms_closed_search” framework, a command-line interface tool for Python on Linux, and its documentation are available for free download at https://github.com/Yura17234/ptms_closed_search (uploaded on 23 June 2025). The pipeline was implemented using several functions from the Pyteomics (version 4.7) Python package [19,20].

### 2.3. Input Files

The input raw LC-MS/MS files of the Orbitrap MS (Thermo Fisher) were converted to .mgf format using the ThermoRawFileParser (version 1.2.0) [21] with default parameters. In order to execute the “ptms_closed_search” pipeline, separate protein sequence files in FASTA format (UP000005640_9606) and a proteome file in query format (UP000005640_9606, 8 July 2025) containing PTMs were downloaded from UniProtKB [22].

### 2.4. “Ptms_Closed_Search” Pipeline

**Figure 1:**
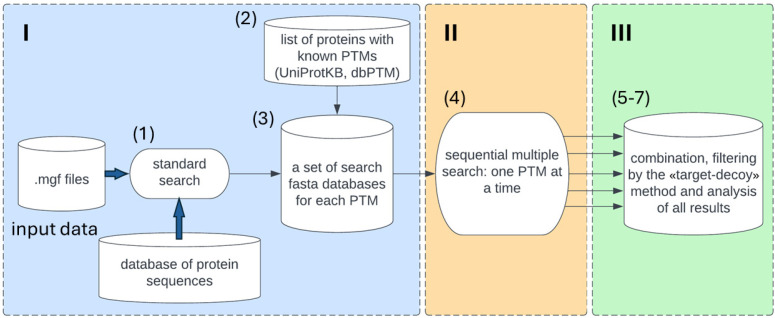
Block scheme of “ptms_closed_search” pipeline. I—preprocessing: conversion of raw files to .mgf files (ThermoRawFileParser) and selection of FASTA file of a proteome; 1—standard MS/MS data search by IdentiPy search engine; 2—automated search of PTMs in public databases; 3—creation of FASTA files for proteins with PTMs. II—multiple searches: 4—sequential searches by IdentiPy search engine with included PTMs as variable modification. III—postprocessing: 5—calculation of PTM-specific FDR based on hyperscore; 6—data filtering by the target–decoy method; 7—combination and visualization of search results.

The algorithm for searching for multiple PTMs (Figure 1) is based on a list of proteins identified in a standard search (standard MS/MS search by database of protein sequences), as well as PTM information from their annotations in the UniProtKB [22] and dbPTM [23] databases. The standard search parameters of IdentiPy (version 0.3.9.6) [24] were the same as the parameters published for each dataset, namely, a database—complete reference human proteome; cleavage enzyme—trypsin with 1 for HEK293 and 2 HeLa and spike-in missed cleavages allowed; oxidation of methionine (M)—variable modification; precursor mass tolerance—5, 6, and 50 ppm for HEK293, HeLa, and spike-in, respectively; and fragment mass tolerance—0.01 Da, 20 ppm, and 0.8 Da for HEK293, HeLa, and spike-in, respectively. The Scavanger (version 0.2.12) [25] tool is used to validate standard search identification results.

The algorithm consists of the following blocks:

#### 2.4.1. Preprocessing (Module I)

1. A list of proteins is generated based on the results of a standard MS/MS search using the IdentiPy [24] search engine and FASTA file for an organism downloaded from UniProtKB. The standard search parameters of IdentiPy were used as the default with the “auto-tuning” of search parameters enabled. Also, parameters for standard MS/MS search were specified according to the dataset description.
2. In accordance with the list of identified proteins (Step 1), the UniProtKB and dbPTM query databases are automatically parsed to annotate a set of PTMs for each protein (Figure S1).
3. The procedure generates a set of FASTA files for each PTM annotated in Step 2. Then, 5000 forward and 5000 reverse random protein sequences are appended to each generated FASTA file. The configuration parameter files for each search are prepared as follows: individual PTMs are designated as variable, and the name of the generated database specified.

Before searching the MS1 and MS2 spectra for modified peptides, the PSMs identified in the standard search were filtered out of the .mgf files.

#### 2.4.2. Multiple Search (Module II)

4. A sequential multiple PTM search was performed using IdentiPy with the “auto-tuning” parameter switched off. The parameters of “precursor mass tolerance” and “fragment mass tolerance” are set based on the optimized value of the precursor mass distribution found in Step 1.

#### 2.4.3. Postprocessing (Module III)

5. The FDR statistic is calculated to evaluate the threshold based on the hyperscore for each modification-specific search at the peptide spectrum match (PSM) using the “target–decoy” method.
6. The results of the individual PTM searches were filtered based on their calculated threshold at 1% of FDR. These individual searches are then merged into a combined PTM result for the analysis.
7. The results of the PTM search are visualized by automatically generating plots in .png format and summary tables in .csv format. These plots and tables display protein coverage, the number of modified peptides and proteins in the sample, and the positions of modifications in protein sequences in .html format.

### 2.5. Data Filtering Pipeline

This method, implemented in the algorithm, calculates FDR statistics using PSMs of unmodified peptides from standard searches and from searches of PTM modified peptides. The data are then filtered based on a propagation error threshold.

**Figure 2:**
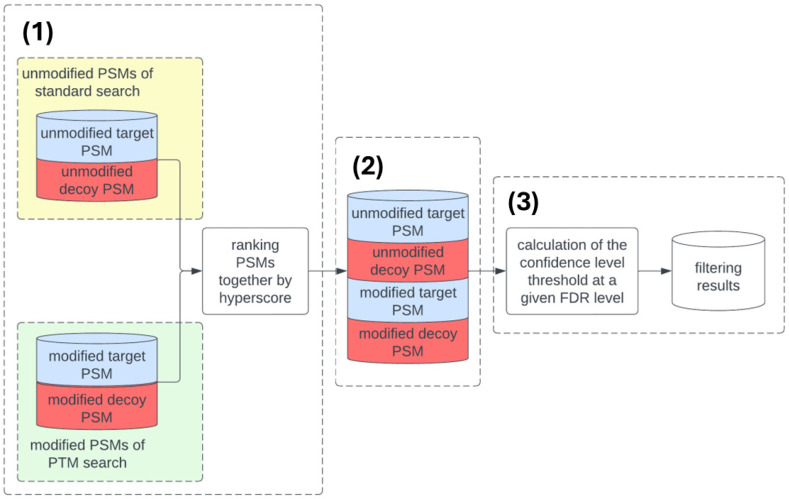
Block scheme of the transferred FDR pipeline calculation: (**1**) combining unmodified and modified PSMs and hyperscore ranking; (**2**) creating target–decoy and modified–unmodified sets of PSMs; (**3**) calculating the transferred FDR.

The main steps of the transferred FDR calculation (Figure 2) are as follows:

1. Unmodified PSMs from the standard search and PSMs with PTMs from multiple searches are merged. All PSMs, both modified and unmodified, are sorted in ascending order by their calculated hyperscore values. For each PSM, a rank is assigned. The first PSM is assigned rank 1, and the last PSM on the list is assigned a rank equal to the total number of identified PSMs.
2. To calculate the PTM-specific FDR, the merged dataset is split into four subsets of unmodified and modified PSMs, as well as target and decoy PSMs.
3. The confidence threshold value at a given FDR level is calculated using the transferred FDR method [11]. This threshold is then used to filter the results of searching for PSMs with PTM.

To increase confidence in the identified modified peptides and ensure the correct FDR calculation, 5000 random target protein sequences (and their 5000 decoys) were added (Figure 1, preprocessing) to each PTM analysis of each FASTA file. The additional random proteins were removed (Figure 2, step 3) from the final results after the threshold value for FDR is determined separately for each modification.

#### 2.5.1. Transferred FDR Calculation

For filtering MS/MS search results, we used a modified transferred FDR approach $\left({\hat{FDR}}_{k}\left(x\right)\right)$ based on the target–decoy method published previously [11]:

(1) $${\hat{FDR}}_{k}\left(x\right)=\frac{N\left(x\right)}{N_{k}\left(x\right)}\cdot {\;\gamma }_{k}\left(x\right)\cdot FDR\left(x\right),$$

where *FDR*(*x*) is the global FDR for all peptide identifications (modified and unmodified) with scores greater than *x*, *N*(*x*) is the number of target identifications without modification with scores greater than *x*, and *N_k_*(*x*) is the number of target identifications of the *k*-modification (e.g., acetylalanine, phosphoserine, etc.) with scores greater than *x*. The parameter *γ_k_*(*x*) is the proportion of *k*-modified peptides (PSM_PTM_) among all false identifications (PSM_PTM_ + PSM_unmodified_).

Instead of using the linear regression model employed by Y. Fu and X. Qian [11], we calculated the parameter *γ_k_*(*x*) using spline-linear regression (Equation (2)):

(2) $$\gamma_{k}\left(x\right)=\left\{\begin{array}{c} a_{1}+b_{1}x,{\;x}_{0}\leq x\leq x_{1}\\ a_{2}+b_{2}x,{\;x}_{1}\leq x\leq x_{2}\\ \ldots \\ a_{n}+b_{n}x,{\;x}_{n-1}\leq x\leq x_{n} \end{array}\right.,$$

where *x*_0_, *x*_1_, …, *x_n_* are the spline nodes, and *a_i_*, *b_i_* are the linear regression coefficients for each interval.

Therefore, the complete equation for rank threshold calculation is as follows:

(3) $${\hat{FDR}}_{m}\left(x\right)=\frac{N\left(x\right)}{N_{m}(x)}\cdot \left\{\begin{array}{c} a_{1}+b_{1}x,{\;x}_{0}\leq x\leq x_{1}\\ a_{2}+b_{2}x,{\;x}_{1}\leq x\leq x_{2}\\ \ldots \\ {\;\;\;a}_{n}+b_{n}x,{\;x}_{n-1}\leq x\leq x_{n} \end{array}\right.\cdot FDR\left(x\right),$$

#### 2.5.2. Error Propagation Calculation for Data Filtering

We noted that as the rank value (hyperscore) increases, the calculated *γ_k_*(*x*) coefficient becomes unstable. This occurs because, as identification quality increases, the number of decoy PSMs decreases. This causes the proportion of modified peptides to false identifications (*γ*-parameter) to vary greatly and significantly deviate from the original trend (see Section 3.3). In order to obtain a stable solution, these data should be filtered out.

To determine the threshold for the rank values at which the data are considered stable, we calculated the error propagation for the *γ*-parameter (Equation (4)). All data with an error propagation value greater than 0.01 were filtered out of the analysis.

(4) $$\sigma \frac{x}{y}=\frac{x}{y}\sqrt{{\left(\frac{\sigma x}{x}\right)}^{2}+{\left(\frac{\sigma y}{y}\right)}^{2}}=\frac{x}{y}\sqrt{{\left(\frac{\sqrt{x}}{x}\right)}^{2}+{\left(\frac{\sqrt{y}}{y}\right)}^{2}}=\frac{x}{y}\sqrt{\frac{1}{x}+\frac{1}{y}},$$

where *x* and *y* are the number of false identifications of modified peptides (PSM_PTM_) and the number of all falsely identified peptides (PSM_PTM_ + PSM_unmodified_), respectively. Assuming a Poisson distribution for both the unmodified and modified decoys, the standard deviations *σx* and *σy* equal $\sqrt{x}$ and $\sqrt{y}$, respectively.

After filtering out data that exceeded the error propagation threshold, subsequent data were extrapolated using an approximation of the last spline segment.

### 2.6. Parameters of MSFragger Search

For the standard open search workflow, the FragPipe (version 23.1) pipeline and MSFragger (version 4.3) [15] were used. The ThermoRawFileParser [21] was used to convert the raw mass spectrometry data into .mzML format with default parameters. The same FASTA databases were used for the analysis as for the CS (UP000005640_9606 and UP000005640_9606, 2025.07.08). For PTM analysis, the open search workflow employed the localization-aware open search (LOS) algorithm in MSFragger with deisotoping, mass calibration, parameter optimization, and monoisotope correction enabled. The analyzed mass range (precursor mass tolerance) was set from −150 to 500 Da. PeptideProphet was applied with the extended mass model and PTM-Shepherd was used for mass shift summarization. The resulting matches were processed by Philosopher (version 5.1.0) [26] and PTM-Shepherd (version 3.0) [27] for validation and mass-shift annotation. The false discovery rate was set to 1%. The fragment mass tolerance was set to 0.01 Da for the HEK293 dataset, 20 ppm for the HeLa dataset, and 0.8 Da for the spike-in dataset. The missed cleavage parameter for MSFragger was set to 1 for HEK293 and 2 for the HeLa and spike-in datasets.

## 3. Results

### 3.1. Standard Search

A database search of all MS/MS data files (24 .mgf files of HEK293 dataset) with IdentiPy (version 0.3.9.6) [24] identified 317,217 PSMs corresponding to 9796 proteins, which is comparable to earlier published results [12]. The overlap of the results by more than 97% led us to conclude that these two methods are comparable, and that this search engine can be used for further algorithm testing.

The same standard search was performed on six .mgf files from the HeLa dataset (PXD002395). A total of 87,868 PSMs corresponding to 6152 proteins were identified. These data overlapped by 95% with previously published results.

### 3.2. Search Space Optimization

**Figure 3:**
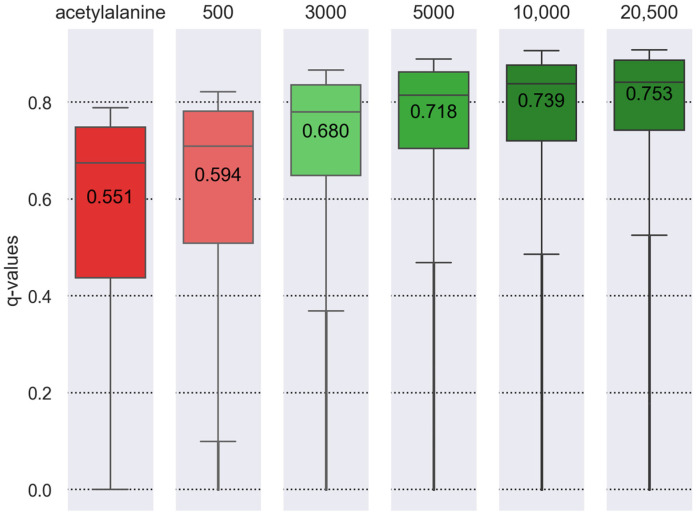
The influence of search database size on the q-value of desired PSMs with PTMs is illustrated using an example of alanine acetylation (HEK293 experiment). The boxplot shows the distribution of q-value scores for each MS/MS search result using FASTA files of various sizes, including an initial search with 959 identified proteins and searches with 500, 3000, 5000, and 10,000 randomly added proteins for the PTM search against the complete proteome of 21,000 proteins. The values in the boxplots represent the mean of the distribution.

First, we evaluated the impact of search space size on the significance of PTM identifications in MS/MS data searches. For the testing, we searched a PTM (e.g., alanine acetylation on HEK293 and serine phosphorylation on HeLa) as a variable modification against the complete reference human protein database (including ~20,500 protein sequences) on 24 (for HEK293) or 6 (for HeLa) .mgf files. After identifying a set of proteins with a PTM, we concatenated this list with an additional set of random proteins to create five different FASTA databases for MS/MS searches. The final number of proteins in a set was 959 (alanine acetylation for HEK293) or 200 (serine phosphorylation for HeLa), with +500, +3000, +5000, or +10,000 random proteins added (Figure 3 and Figure S2). To estimate the significance of the identifications, we calculated the q-value [28] as the ratio of the number of decoys to the number of target PSMs with PTM. Please note that we used information from added random proteins for this FDR calculation: the number of the target and decoy PSMs with PTMs.

The distribution of q-values of target PSMs against the complete proteome was used as a reference for the MS/MS search. The addition of 500 random proteins did not result in a significant change in the distribution of q-values when compared to the database that was generated only by a single PTM. Conversely, the addition from 3000 to 10,000 proteins demonstrated a comparable distribution of q-values to the level of confidence for the entire proteome.

**Figure 4:**
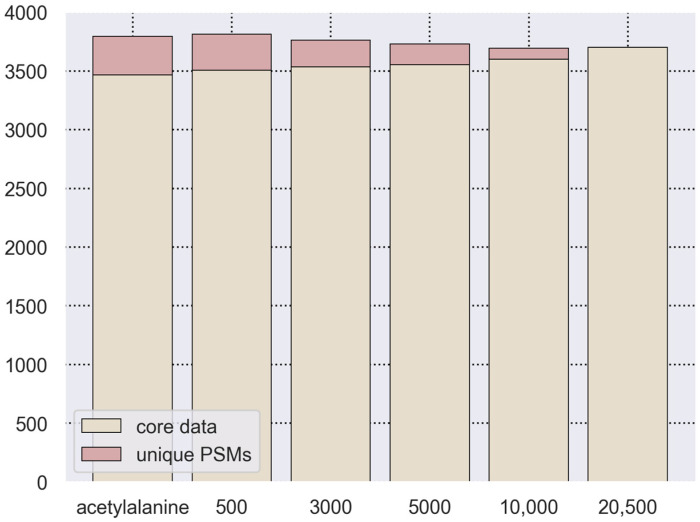
Bar chart of the number of target acetylated alanine PSMs with q-value ≤ 0.05. Core data—PSMs identified in the full proteome search and searches with added random proteins; unique PSMs—PSMs identified only in the corresponding search.

The results of these searches were filtered to determine the most significant identifications for q-values ≤ 0.05 (Figure 4 and Figure S3). The number of PSMs from each search was compared with those PSMs searched against the full proteome database. Core PSM identification (data found for all searches) was performed using a unique identifier (primary key) consisting of the file name, spectrum scan number, and modified peptide sequence. As illustrated, the vast majority of reliable identifications (q-value ≤ 0.05) were identified in each search regardless of the search space.

### 3.3. Transferred FDR and Error Propagation Data Filtering

**Figure 5:**
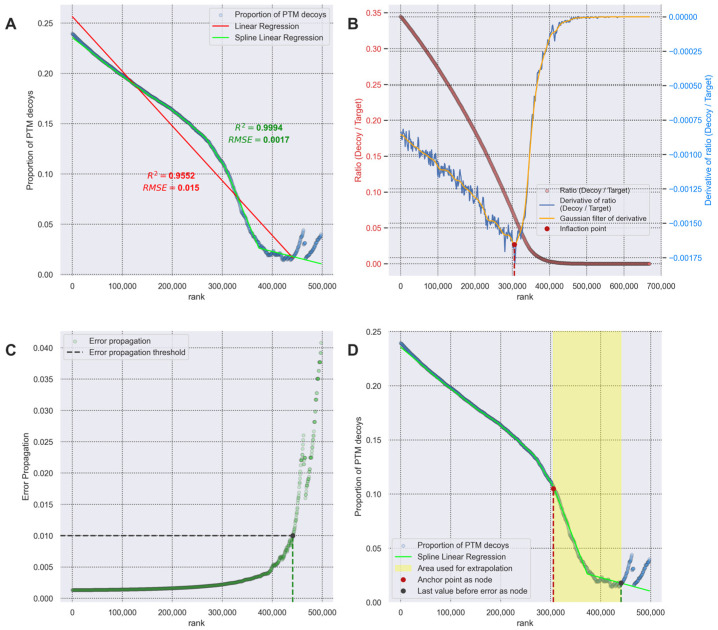
An example of the approximation of the *γ_k_*(*x*) parameter for alanine acetylation and its extrapolation at high ranks. The identified PSMs were ranked according to their calculated hyperscores (the higher the rank, the more significant the identification). (**A**) A comparison of linear and spline-linear regression approximation algorithms (Equation (2)). (**B**) Determination of region where the number of target PSMs exceeds the number of decoy PSMs. In red is the ratio of the number of all decoy PSMs to the number of all target PSMs. In blue is the derivative of this ratio and the Gaussian filter of the derivative (orange). The inflection point of the derivative is used for node spline rooting. (**C**) Error propagation values for each threshold are calculated according to the Equation (4). (**D**) Approximation of *γ_k_*(*x*) parameter by spline-linear regression with a calculated rooted spline node and an error propagation threshold implemented in the “ptm_closed_search”. The yellow rectangle shows the main approximation area, which is used for extrapolating *γ_k_*(*x*) at high ranks.

In our study, we compared linear regression [11] and spline-linear regression in approximating the proportion of falsely identified modified peptides to all PSMs in the transferred FDR (Figure 5A). The R^2^ and RMSE parameters show that the spline-linear function provides more accurate data description (R^2^ 0.9994, RMSE 0.0017) compared to linear regression approximation (R^2^ 0.9552, RMSE 0.015). For this reason, we used a spline-linear approximation in the threshold calculation method (Equation (3)).

We saw that the data for the gamma parameter were nonlinear in areas where there were more target PSMs than decoy PSMs (Figure 5A, blue dots on the right side of the graph). In this area, randomly rooting a spline node in a place where the variability is different can cause the spline segment to fluctuate significantly. To reduce this variability, we created a rule for defining spline nodes. The reference point should be an inflection point for one of the nodes in the linear regression spline. To determine the inflection point, we used the ratio of the number of decoy PSMs to the number of target PSMs (Figure 5B). The inflection point (dotted red line in Figure 5B,D) was calculated as the global minimum of the first derivative function (blue line, Figure 5B) of the ratio.

To filter out the unstable data on the high rank before the approximation of the proportion of falsely identified modified peptides, the error propagation was calculated according to Equation (4) (Figure 5C). All data exceeding the 0.01 error propagation threshold were excluded from further FDR calculation (yellow rectangle, Figure 5D). For the transferred FDR calculation of the high rank, we use the extrapolated data from the last segment of the spline-linear regression anchored by the calculated inflection point.

### 3.4. Testing the Algorithm on HEK293 LC-MS/MS Data

The algorithm, running on 10 CPU threads (3.2 GHz), completed a comprehensive PTM analysis on the HEK293 (see Section 2) test dataset in 4.8 h. It identified 9340 PSMs and 3528 modified peptides corresponding to 1678 proteins at a 1% FDR level. These modified proteins accounted for approximately 18.2% of the proteins obtained from the standard search. The algorithm tested the data for 87 PTMs across various amino acids. Testing of the pipeline was based on information from UniProtKB in query format (UP000005640_9606, 2025.07.08 version) and dbPTM (2025.01.20 version).

A search analysis of the test data identified 13 protein modifications (Figure S4). The following PTMs and their number of PSMs were identified: phosphoserine—2158; acetylalanine—1866; acetylmethionine—883; acetylserine—819; dimethylarginine—534; phosphothreonine—440; methylarginine—329; acetyllysine—202; citrullinearginine—196; acetylthreonine—124; trimethyllysine—121; deamidated asparagine—79; phosphotyrosine—32.

We also ran a test PTM search on the HeLa dataset (PXD002395), which took 3.5 h. A total of 1196 PSMs and 660 modified peptides corresponding to 429 proteins were identified at a 1% FDR level. According to the analysis results, 6.97% of proteins contain PTMs.

At the PSM level, 15 PTMs were identified in the HeLa dataset (Figure S5), for example: phosphoserine—316; acetylalanine—171; acetylmethionine—115; trimethyllysine—93; dimethyllysine—85; deamidated asparagine—70; methyllysine—66; dimethylarginine—66; phosphothreonine—54; methylarginine—43; acetylthreonine—17; citrullinearginine—15.

#### 3.4.1. Transferred FDR and Separate FDR Comparison

**Figure 6:**
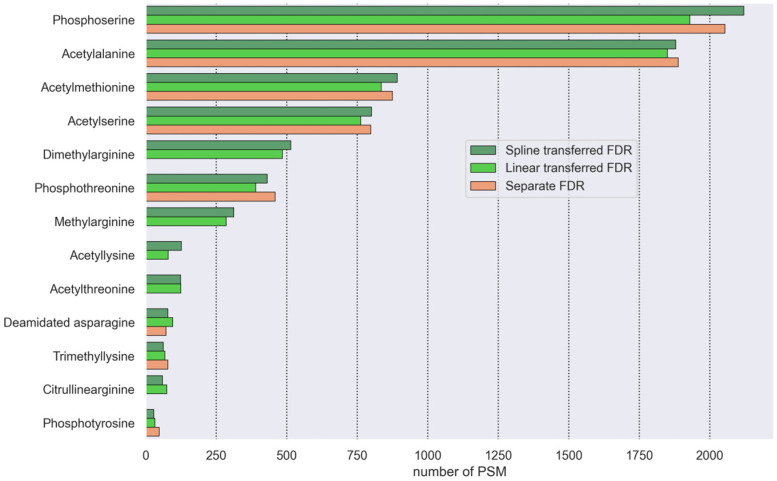
Comparative analysis of data filtering algorithms. The results for spline transferred FDR, linear transferred FDR, and separate FDR for individual PTMs. Note that for some PTMs, only the spline- and linear-transferred FDRs give the results above the significance threshold (HEK293 dataset, PXD001468).

By comparing the number of PSMs (Figure 6) and proteins (Figure S5) for each filtered PTM, we confirmed that the linear and the spline transferred FDR methods implemented in the algorithm identify a similar number of PTMs to the separate FDR method. This indirectly suggests that the filtering of identified PSMs by our modified FDR estimation is correct. Additionally, our method and the linear transferred FDR can identify modifications that did not pass the separate FDR filtering method. These include citrullination of arginine, acetylation of lysine and threonine, and methylation of lysine and arginine.

#### 3.4.2. Coverage Increase by Modified Peptide Search

**Figure 7:**
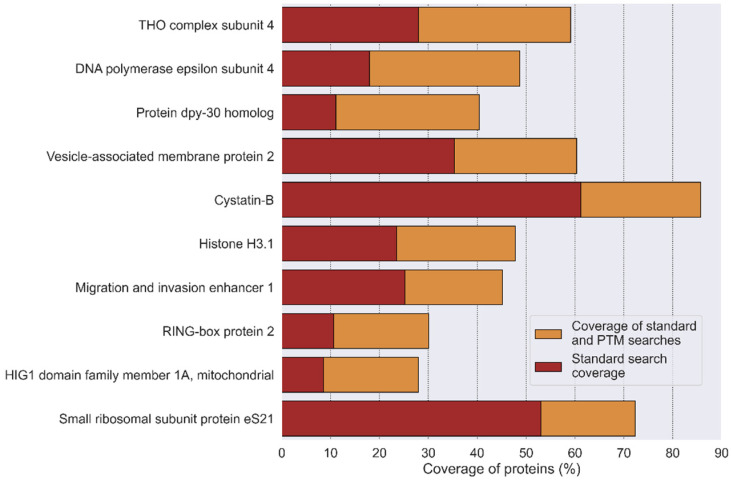
The coverage results of the top ten proteins by the standard search (in red) and after the “ptms_closed_search” (in orange) algorithms. The data are listed in order from the most to the least coverage (HEK293 dataset, PXD001468).

This algorithm enables the detection of additional modified peptides, thereby increasing the overall protein sequence coverage from an average of 30.8% to 33.5% (Figure 7) and improving the significance of protein identification.

#### 3.4.3. PTM Site Localization

After processing the results, the algorithm displays all identified and localized PTMs on protein sequences for better representation and visualization. Fixed modification of cysteine (carbamidomethylation) and oxidation of methionine as variable modification were excluded from further analysis. The result is saved as an .html file in the analysis results directory (Figure S7).

The graphical output of the script includes the number of modified PSMs and a list of the identified peptides and proteins for each PTM in the sample (Figures S4 and S5). The output also includes the calculation of protein coverage (Figure 7) after the PTM search and visualization of PTM localization sites, as described above. Together, this result of analysis provides a clear picture of the PTMs and a variety of proteoforms in the sample.

### 3.5. Comparison of “Ptms_Closed_Search” on Truncated and Full Databases

To reduce time without loss of information, we proposed and implemented the idea of a closed search algorithm for PTM on a truncated protein database constructed on information from UniProt and dbPTM—the fast search mode. The pipeline also includes a full search mode that performs searches against the entire protein database for each modification.

We compared the number of identifications in fast search and full search modes with the aim of assessing the completeness of PTM annotation in public databases. These comparisons were only performed at the PSM level. Sets of PSMs with the same modification type were compared, and different localizations of the same modification were ignored (e.g., acetylalanine and acetylserine will be the same modification type—acetyl). PSMs were intersected by MS/MS file name, scan number, charge, and peptide sequence.

**Figure 8:**
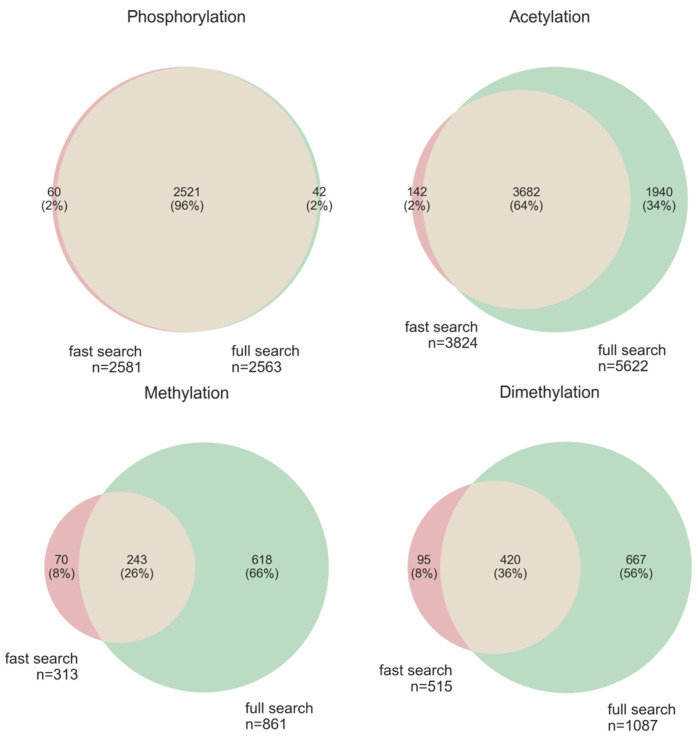
The results of the “ptms_closed_search” identification of PTMs in the HEK293 dataset (PXD001468) in two regimes: fast search mode (truncated database) in red and full search mode (complete human proteome) in green. PSMs of these two search modes were intersected based on the MS/MS file name, scan number, charge, and peptide sequence.

The results of this comparison (Figure 8) showed that, for well-annotated PTMs, such as phosphorylation, the results of these two regimes do not differ. However, for the less annotated PTMs, the results of the search against the full database can increase the number of PSMs by more than two times. Therefore, for the remaining comparisons, we decided to use a full database search.

#### 3.5.1. Comparison of Closed Search (“ptms_closed_search”) to Open Modification Search (“MSFragger”)

We compared two different approaches in PTM search CS (“ptms_closed_search” by IdentiPy engine) and OMS (“MSFragger”) (Figure S8). We also filtered out mass shifts in PSMs, which were annotated by the PTM-Shepherd (FragPipe pipeline) as unannotated mass shifts.

**Figure 9:**
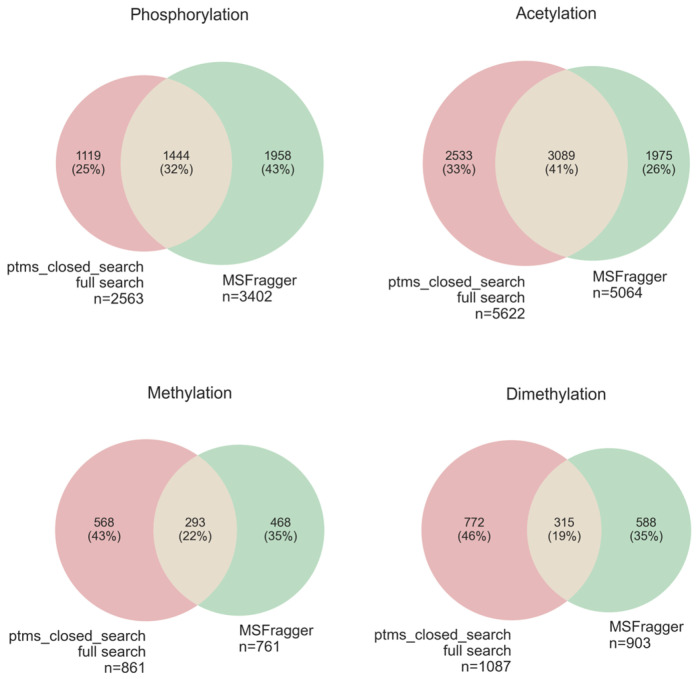
The results of the identification of PTMs in the HEK293 dataset (PXD001468) by “ptms_closed_search” in full search mode (complete human proteome) in red and “MSFragger” (FragPipe pipeline) in green. PSMs of these two search engines were intersected based on the MS/MS file name, scan number, charge, and peptide sequence.

Our results showed that the two engines identified about 30% of PSMs as consolidated discovery, leaving about one-third of unique PSMs for each search engine (Figure 9). Since there is no commonly accepted metric to directly compare the results, the outcomes of these search engines should be reviewed separately. We provide the researchers with annotated fragmentation spectra in graphical form (Figure S9). Note that open search approaches do not identify the location of PTMs on the sequence of the peptide; these data should be treated as guidelines for the discovery of PTMs. Additionally, we suggest combining these methods to increase the number of PTM identifications, as recommended previously [12].

#### 3.5.2. Sensitivity Comparison of CS (“Identipy”) and OMS (“MSFragger”) on 20 Spike-In Ubiquitinated Proteins

**Figure 10:**
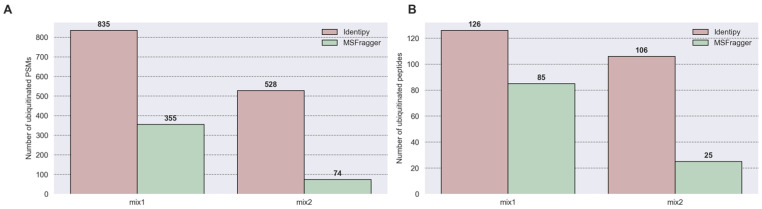
The number of identifications of ubiquitinated peptides within the spike-in experiment. Twenty ubiquitinated proteins were added to the proteome of HEK293 cells (MassIVE 506 MSV000088971) in amounts of 2 pmol in mix1 and 1 pmol in mix2. The search for sensitivity comparison was performed with CS (“IdentiPy”) and OMS (“MSFragger”) approaches. (**A**) At the PSM level. (**B**) At the level of unique peptides identified.

To compare and validate the sensitivity and accuracy of closed and open search approaches for individual PTMs, a test search was performed on data with known concentrations of ubiquitinylated proteins. A sensitivity assessment of our pipeline revealed (Figure 10) that IdentiPy identifies 2.3 times more PSMs and 1.5 times more unique peptides than MSFragger at a concentration of 2 pmol (mix1). At a concentration of 1 pmol (mix2), IdentiPy identifies 7.1 and 4.2 times more PSMs and unique peptides than MSFragger, respectively. An assessment of the accuracy of the method, defined as the ratio of the delta in the number of PSMs at high and low concentrations to the difference in concentrations, showed that our method is comparable in accuracy to open search.

## 4. Discussion

In this manuscript, we present an algorithm that enables us to perform a sequential closed search for modified proteins. This approach is based on the one-by-one [8] and cascaded [10] search algorithms, which increases the sensitivity of identifying peptides with PTM. As previously demonstrated [29], a sequential closed PTM search solves the problem of combinatorial explosion by considering all variants of modified peptides during the search but introduces the issue of calculating PTM-specific FDR. We should emphasize that the hyperscores are calculated independently for each individual PSM based on the number of matched fragment ions and their intensities. It is an absolute value, regardless of whether the peptide is modified or unmodified, and irrespective of the composition of the sample as a whole. In our approach, the hyperscore is used solely as a ranking metric for ordering PSMs before calculating the transferred FDR. This approach does not require matching or normalizing score distributions across different subsets (hypersore distributions are shown in Figure S10). Instead, it relies on the comparability of ranks.

There are several limitations to consider when conducting proteomics experiments, of which the major ones include a loss of spatial and subcellular distribution in protein abundances, incompleteness of the information about the proteome as such due to the limited dynamic range of MS instruments, redundancy in isoforms and splice variant assignment, and loss of proteoform annotation with the bottom-up approach due to conversion of proteins into peptides. Furthermore, it is difficult to quantify proteins in a shotgun experiment, which is usually based on only three to four peptides. These limitations are usually well known within the MS community working with protein samples [2].

In this paper, during the development of a new algorithm for PTM annotation, all above-mentioned limitations were not considered. Most researchers conducting mass spectrometry studies and spectra annotation using closed search use a limited set of PTMs as variables by default—usually methionine oxidation and N-terminal acetylation. In our work, we propose conducting an additional search for information from public databases.

In the current version, only single modifications (plus methionine oxidation) were considered in MS/MS searches. Peptides carrying multiple modifications (except for methionine oxidation) will not be found. We see two ways to solve this problem. The first approach is to create databases of poly-modifications (double, triple, etc.) based on the frequency of occurrence of these modifications in the Uniprot and dbPTM databases. Based on the tryptic model of the proteome, a database of peptides is created, and the frequencies of occurrence of these modifications are calculated, as well as lists for subsequent search. The second approach consists of an initial search for PTM annotation of peptides using an open search method. After that, the list of modification combinations can be used as a basis for a closed search. The second approach can be used in combination with the first to create a comprehensive list of modifications.

As has been described previously [30], a reduction in search space improves the sensitivity of PSM identification. We also tested the impact of search database size on the distribution of q-values for target PSMs. As demonstrated in Figure 3 and Figure 4 (Figures S2 and S3), the distribution of the q-values of PSMs is comparable when searching for modified peptides from a database of protein sequences comprising 3000 and 10,000 proteins. A further decrease in search database size distorts the distribution and increases the number of false-positive identifications (Figure 4 and Figure S3). We determined that the optimal database size is 5000 protein sequences. This size does not alter the distribution of q-values as well as the number of false identifications while requiring less computing time. Therefore, for each PTM search, we constructed a specific set of protein sequences based on information from the UniProtKB and dbPTM databases and also added a list of random proteins. If a given PTM’s protein database contained fewer than 5000 proteins, these datasets were supplemented with an additional 5000 random proteins.

Several approaches have been described previously to calculate FDR for PTM identifications: separate FDR and transferred FDR [11]. During the data analysis process, we noted that the proportions of modified decoys do not behave linearly: they are poorly approximated by the linear regression proposed in the original manuscript [11]. Therefore, we modified the transferred FDR algorithm by improving the approximation model to a more accurate spline-linear regression method. This resulted in a superior description of the proportion of modified decoys compared to the original linear approximation. The superiority of the spline model (Equation (2)) is evident from the R^2^ and RMSE values (Figure 5A), whereas the linear regression achieves less accurate results.

To account for the ratio of decoy to target PSMs, we introduced a rule designating the inflection point as the anchor node for the spline-linear regression model. We observed that, as the rank increases, the first derivative of the ratio changes sign. To determine this inflection point, which we propose as a stable node for approximation, we introduced Gaussian data smoothing. Identification of this point is performed by calculating the global minimum of the smoothed derivative.

As described previously [11], as the PSM score increased, the data became unstable, introducing error into the approximation. We also observed this behavior in our data. To address this issue, we applied error propagation filtering and defined a cutoff point at which the error exceeded 0.01. The proportion of PTM decoys above this threshold was determined by extrapolating the last segment of the spline-linear regression.

We also encountered an uneven distribution of hyperscores in bins of PSMs in FDR estimation. Applying PSM ranking solved this problem. Furthermore, this ranking allows for cross-platform comparisons of search results obtained using different scoring function calculation algorithms.

As noted by Y. Fu and X. Qian [11], a major limitation of separate FDR is that its calculation is based only on the small subset of PSMs with a given modification. This often provides too few decoys for reliable statistics. This leads to unstable or overly conservative FDR values, causing many true modified identifications to be discarded. Transferred FDR addresses this problem by leveraging global FDR statistics, modeling the relationship between global and subgroup decoys, and extrapolating for high-scoring PSMs. As a result, this approach yields more accurate and stable estimates even for rare modification groups, allowing more PSMs and PTMs to pass filtering in our pipeline compared to separate FDR.

As we showed when comparing the performance of “ptms_closed_search” in fast search and full search modes, fast search mode is sufficient when there are enough annotations in the databases (e.g., for phosphorylation). However, for modifications that are still poorly represented in the Uniprot and dbPTM databases, a search should be performed against the entire proteome database of the organism.

One alternative approach is to use the search data from the OMS algorithm to compile lists of proteins and modifications based on its search results. However, as we have shown in our work, this approach also has its drawbacks. Open search has lower sensitivity, which can lead to an underestimation of modifications for specific proteins in a matrix of complex mixtures.

The size of protein databases for searching not only affects the quality of FDR calculations but also influences the sensitivity of the method, namely the number of detected PTM proteins. We found that using a closed search in full search mode gives guaranteed, comparable results in terms of the number of PSMs identified with OMS. At the same time, as discussed above, a closed search is more sensitive than the OMS approach.

To confirm the latter statement, we performed sensitivity and accuracy analyses on spike-in data with known concentrations of proteins with ubiquitination. Unsurprisingly, the closed search method yielded more than twice as many identifications, while the accuracy of the methods was comparable.

Thus, we can conclude that closed search is capable of providing substantial additional information, and that further development of closed-search PTM optimization methods is warranted. In our pipeline, we have already implemented the possibility to supply additional lists of proteins (which are unambiguously required to be checked for modification) as input for each PTM. This additional list may serve as a basis for integrating our “ptms_closed_search” pipeline with the results of open search. To achieve this, it will be necessary to implement a converter that transforms “MSFragger” search results into modification-specific protein lists to be used in the targeted PTM search, which may constitute a subject for future work.

## 5. Conclusions

We demonstrated that our method can perform multiple PTM searches using various optimization techniques and improved data filtering. This allows us to obtain extra information about PTMs of proteins identified in a sample, compared with previously developed algorithms. We believe that our pipeline improvements will enable faster and more accurate PTMs screening, providing a deeper understanding of the proteoform spectrum in a sample. These improvements will also enable comparison and data combination with other MS search methods. In future work, the algorithm can be further improved by incorporating information from open modification searches to guide targeted closed searches, enabling more comprehensive and sensitive identification of complex and low-abundance PTMs.

## Data Availability

The dataset was generated from HEK293 human cell lysates [12]. The 24 raw files, as well as peak lists and spreadsheets, were downloaded from ProteomeXchange (accession number PXD001468). Details on sample preparation and LC-MS/MS protocols can be found in the original manuscript [12], available at https://www.ebi.ac.uk/pride/archive/projects/PXD001468 (accessed on 3 March 2025). The HeLa cell lysate dataset used for validation was obtained from the PRIDE repository (ProteomeXchange accession number PXD051723, accessed on 11 December 2025) [17]. The dataset consists of six raw LC–MS/MS files generated from HeLa cells and analyzed using an Orbitrap mass spectrometer. Detailed information on sample preparation, liquid chromatography, and mass spectrometry acquisition parameters is available in the original publication [17]. The spike-in benchmark dataset used for sensitivity and accuracy evaluation of ubiquitination analysis was obtained from the MassIVE repository (accession number MSV000088971, accessed on 12 December 2025) [18]. This dataset includes HEK293 cell lysates supplemented with known concentrations of ubiquitinated proteins and analyzed by LC–MS/MS on an Orbitrap Lumos mass spectrometer. Experimental design and acquisition details are described in the corresponding original study [18].

## Supplementary Materials

The following supporting information can be downloaded at: https://www.mdpi.com/article/10.3390/proteomes14010007/s1, Figure S1: The list of PTMs created based on information from the UniProtKB and dbPTM databases. A list of protein identifications was generated using a standard MS/MS search of the HEK293 dataset (PXD001468) (For the details see Section 2.4); Figure S2: The influence of search database size on the q-value of desired PSMs with PTMs is illustrated using an example of serine phosphorylation (HeLa dataset, PXD002395). The boxplot shows the distribution of q-value scores for each MS/MS search result using FASTA files of various sizes, including an initial search with 200 identified proteins and searches with 500, 3,000, 5,000, and 10,000 randomly added proteins for the PTM search against the complete proteome of 21,000 proteins. The values in the box plots represent the mean of the distribution; Figure S3: Barplot of the number of target phosphorylated serine (HeLa dataset, PXD002395) PSMs with q-value ≤ 0.05, Core data—PSMs identified in full proteome search and searches with added random proteins, unique PSMs—PSMs identified only in corresponding search; Figure S4: The number PSMs (A), peptides (B) and proteins (C) for top 13 different PTMs of proteins identified by «ptms_closed_search» algorithm of the HEK293 dataset (PXD001468); Figure S5: The number PSMs (A), peptides (B) and proteins (C) for top 15 different PTMs of proteins identified by «ptms_closed_search» algorithm of the HeLa dataset (PXD002395); Figure S6: Comparison of the number of proteins filtered by Spline transferred FDR (A), separate FDR (B) и Linear transferred FDR (C); Figure S7: A print screen of results visualization of “ptms_closed_search” algorithm output (.html format) with PTM sites localization on sequences of individual proteins, the oxidation of methionine is not taken into account when describing modifications. The modification positions are listed in square brackets; Figure S8: Unmodified PSMs identified by IdentiPy and MSFragger; Figure S9: Examples of MS/MS spectra annotation of HEK293 dataset (PXD001468) identified by “ptms_closed_search” by IdentiPy search engine; Figure S10: Distribution of all unmodified decoy and target PSMs, as well as decoy and target PSMs with PTM, without filtering.

## Abbreviations

The following abbreviations are used in this manuscript:

PTM	Post-translational modification
OMS	Open modification search
CS	Closed search
FDR	False discovery rate
PSM	Peptide spectrum match
