# Supplementary material for: PTMs_Closed_Search: Multiple Post-Translational Modification Closed Search Using Reduced Search Space and Transferred FDR
Source: Proteomes. 2026 Feb 2;14(1):7. doi: 10.3390/proteomes14010007 (PMC12921745; doi:10.3390/proteomes14010007)
Supplement: Supplementary file 1 [file proteomes-14-00007-s001.zip › proteomes-3970048-supplementary.pdf]

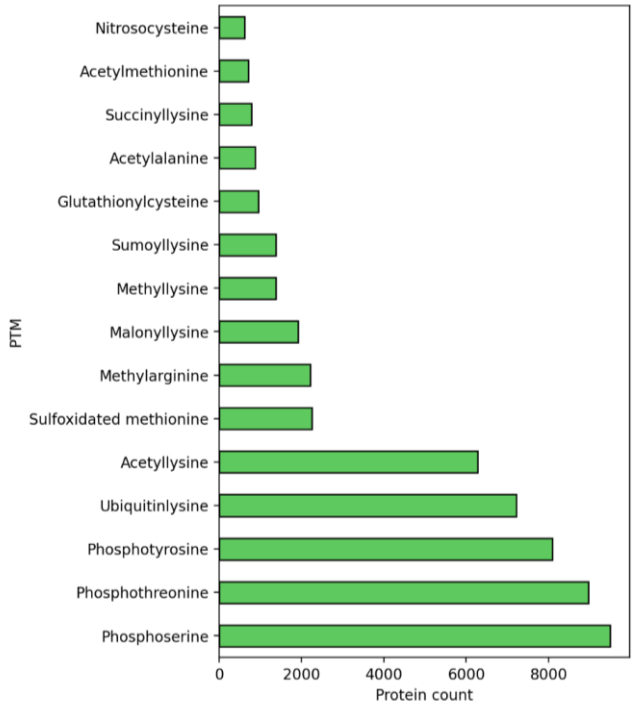

Figure S1. The list of PTMs created based on information from the UniProtKB and dbPTM databases. A list of protein identifications was generated using a standard MS/MS search of the HEK293 dataset (PXD001468) (For the details see section M&M 2.4).

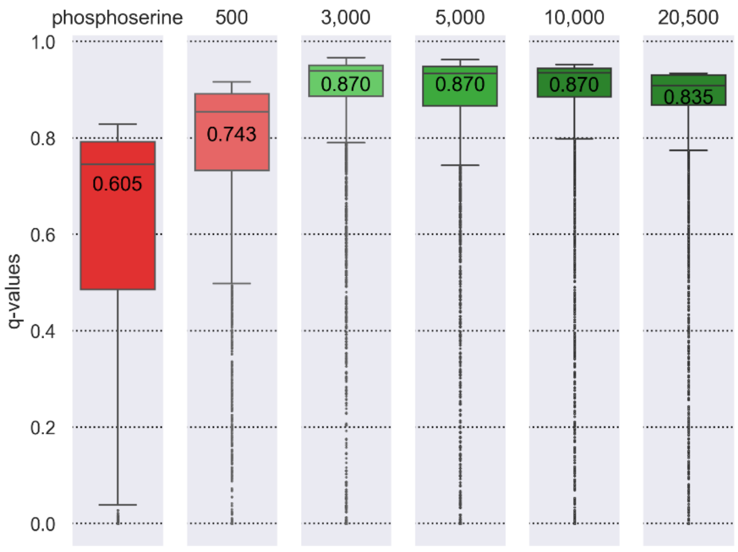

Figure S2. The influence of search database size on the q-value of desired PSMs with PTMs is illustrated using an example of serine phosphorylation (HeLa dataset, PXD002395). The boxplot shows the distribution of q-value scores for each MS/MS search result using FASTA files of various sizes, including an initial search with 200 identified proteins and searches with 500, 3,000, 5,000, and 10,000 randomly added proteins for the PTM search against the complete proteome of 21,000 proteins. The values in the box plots represent the mean of the distribution.

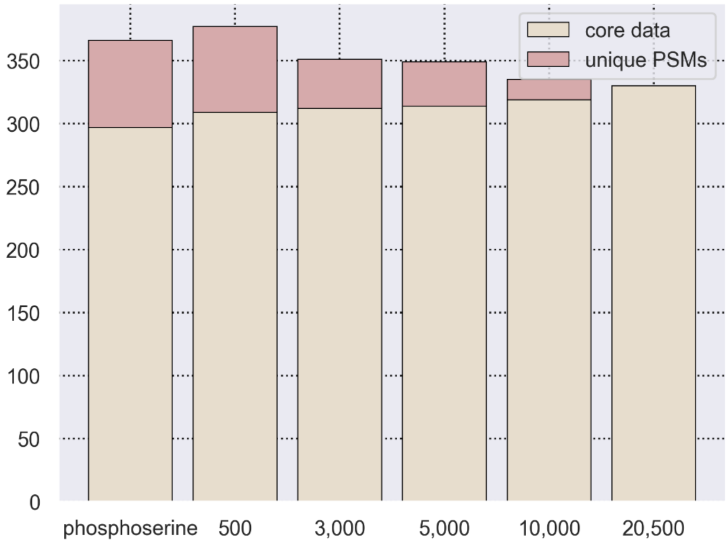

Figure S3. Barplot of the number of target phosphorylated serine (HeLa dataset, PXD002395) PSMs with q-value ≤ 0.05, Core data – PSMs identified in full proteome search and searches with added random proteins, unique PSMs – PSMs identified only in corresponding search.

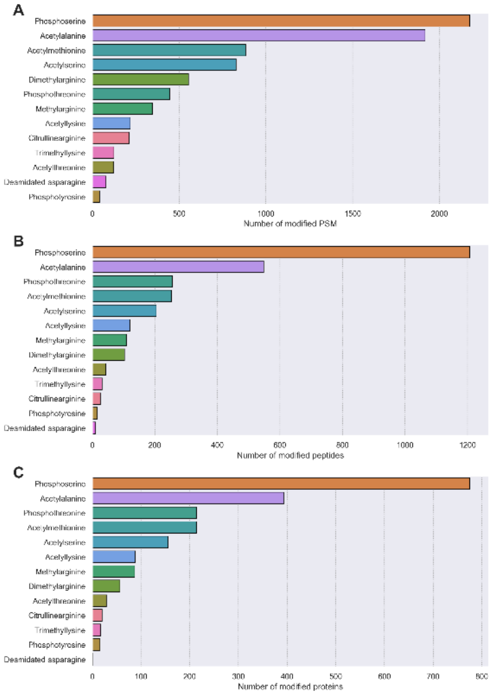

Figure S4. The number PSMs (A), peptides (B) and proteins (C) for top 13 different PTMs of proteins identified by «ptms_closed_search» algorithm of the HEK293 dataset (PXD001468).

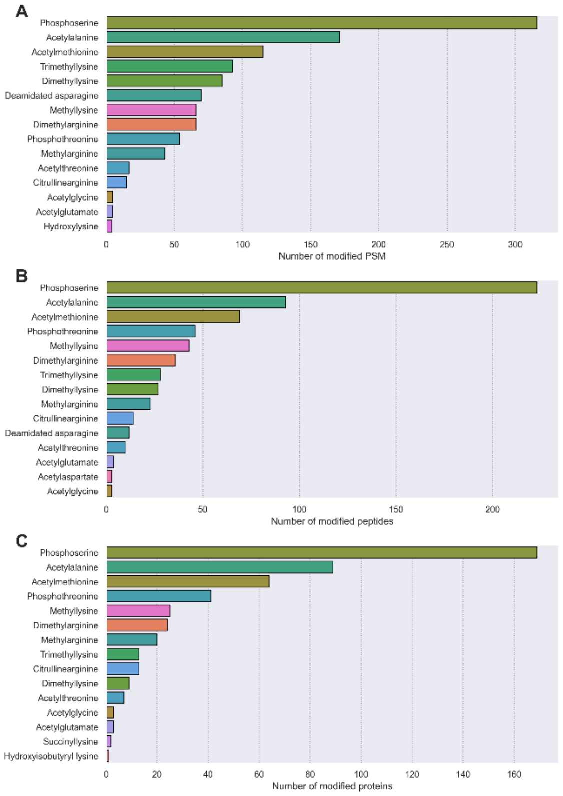

Figure S5. The number PSMs (A), peptides (B) and proteins (C) for top 15 different PTMs of proteins identified by «ptms_closed_search» algorithm of the HeLa dataset (PXD002395).

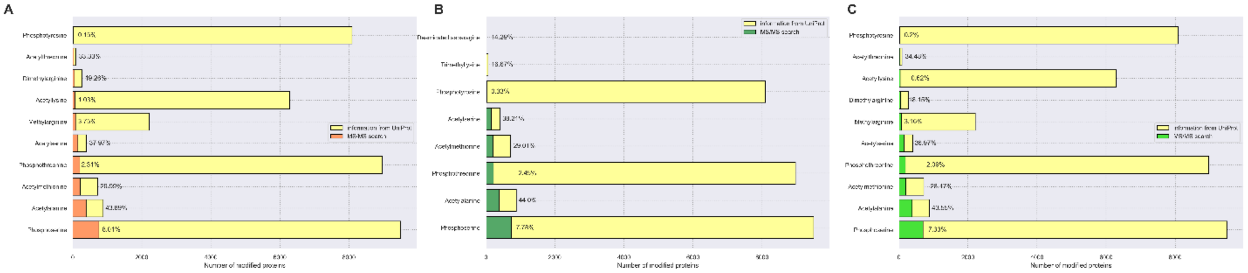

Figure S6. Comparison of the number of proteins filtered by Spline transferred FDR (A), separate FDR (B) и Linear transferred FDR (C)

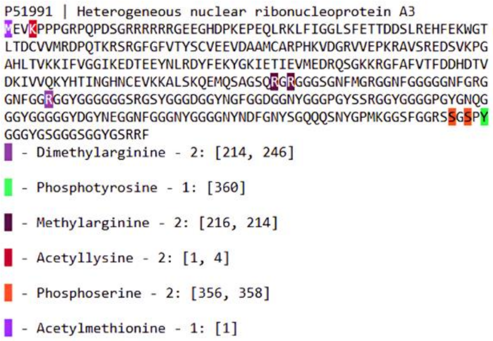

Figure S7. A print screen of results visualization of "ptms_closed_search" algorithm output (.html format) with PTM sites localization on sequences of individual proteins, the oxidation of methionine is not taken into account when describing modifications. The modification positions are listed in square brackets.

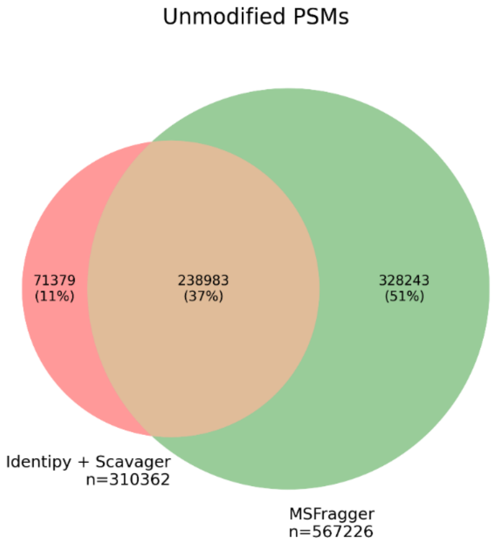

Figure S8. Unmodified PSMs identified by IdentiPy and MSFragger

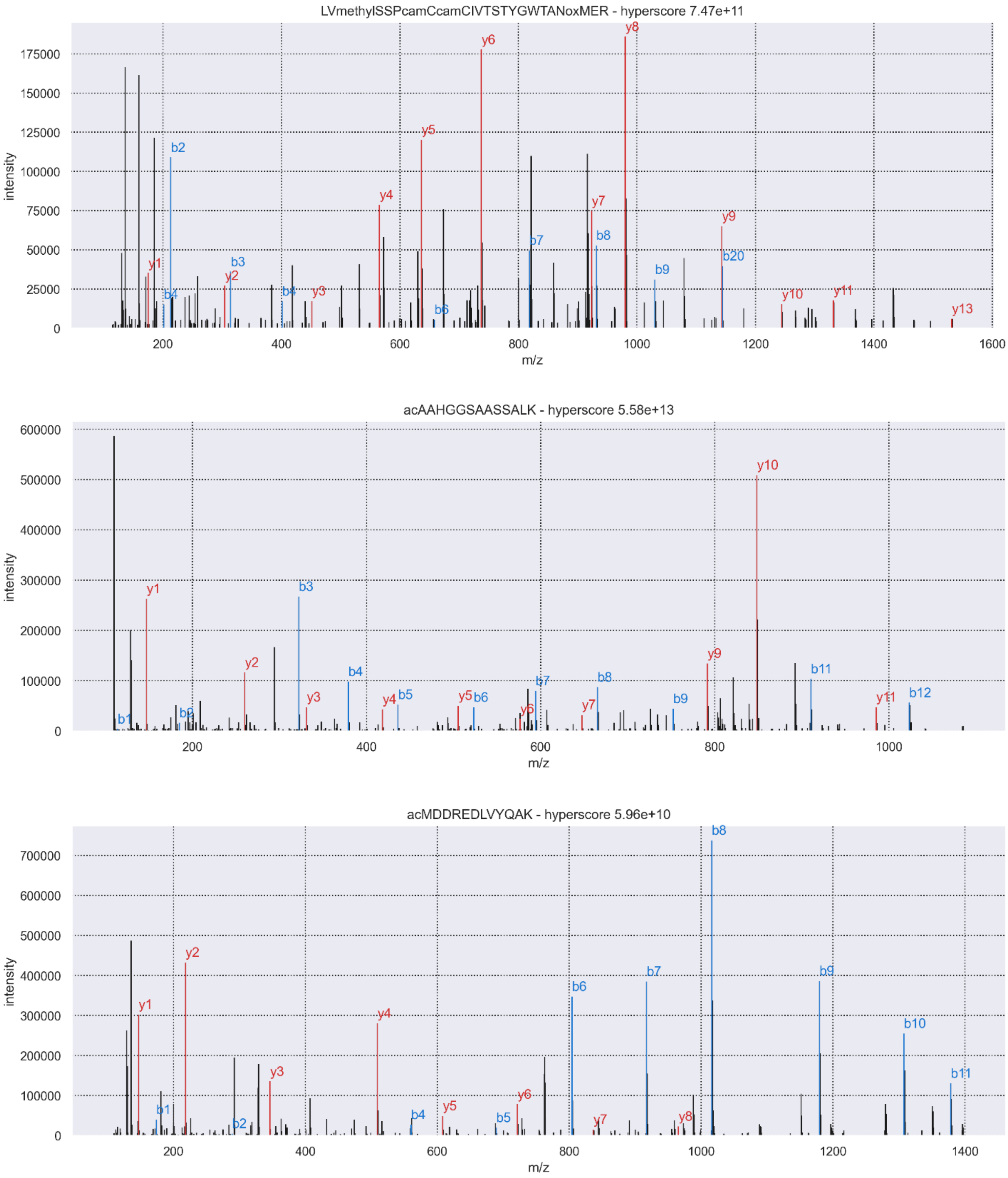

Figure S9. Examples of MS/MS spectra annotation of HEK293 dataset (PXD001468) identified by “ptms_closed_search” by IdentiPy search engine

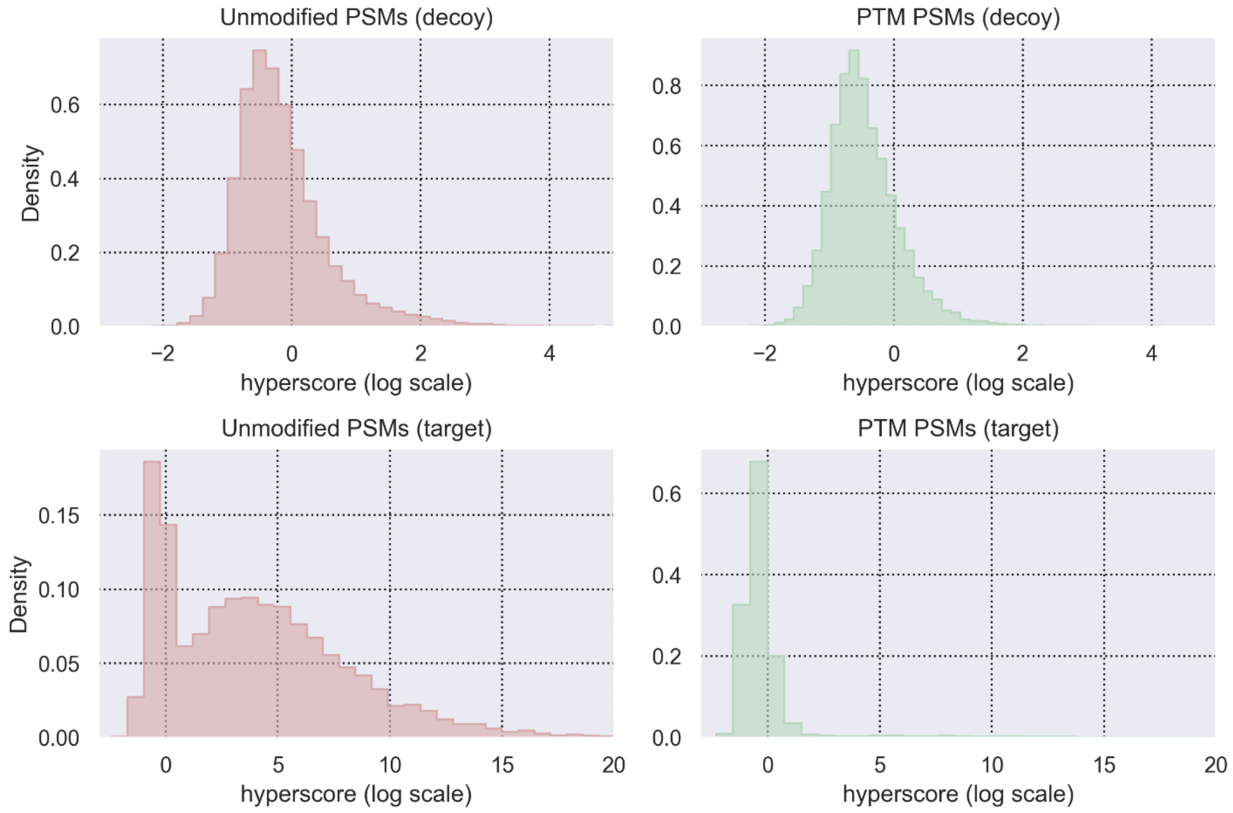

Figure S10. Distribution of all unmodified decoy and target PSMs, as well as decoy and target PSMs with PTM, without filtering.
